# Comparison of Time-Zero Primary Stability Between a Biodegradable Magnesium Bone Staple and Metal Bone Staples for Knee Ligament Fixation: A Biomechanical Study in a Porcine Model

**DOI:** 10.1177/23259671241236783

**Published:** 2024-03-25

**Authors:** Adrian Deichsel, Johannes Glasbrenner, Michael J. Raschke, Matthias Klimek, Christian Peez, Thorben Briese, Elmar Herbst, Christoph Kittl

**Affiliations:** * Department of Trauma, Hand and Reconstructive Surgery, University Hospital Münster, Münster, Germany; Investigation performed at the Department of Trauma, Hand and Reconstructive Surgery, University Hospital Münster, Münster, Germany

**Keywords:** biomechanics, bone staples, cortical fixation, ligament reconstruction, magnesium

## Abstract

**Background:**

Bone staples have been shown previously to be a viable modality for cortical tendon graft fixation in ligament knee surgery. However, soft tissue reactions have been reported, making implant removal necessary. Magnesium alloys are a promising material for biodegradable orthopaedic implants, with mechanical properties closely resembling those of human bone.

**Purpose:**

To compare the primary stability of a biodegradable bone staple prototype made from magnesium to bone staples made from metal in the cortical fixation of tendon grafts during knee surgery.

**Study Design:**

Controlled laboratory study.

**Methods:**

Primary stability of peripheral tendon graft fixation was assessed in a porcine model of medial collateral ligament reconstruction. Two commercially available metal bone staples (Richards fixation staple with spikes [Me1] and spiked ligament staple [Me2]) were compared with a magnesium bone staple prototype for soft tissue fixation. Primary stability was assessed using a uniaxial materials testing machine. Cyclic loading at 50 and 100 N was applied for 500 cycles each, followed by load-to-failure testing.

**Results:**

After 500 cycles at 50 N, elongation was 1.5 ± 0.5 mm in the Me1 group, 1.9 ± 0.5 mm in the Me2 group, and 1.8 ± 0.4 mm in the magnesium group. After 1000 cycles of loading (500 cycles at 50 N and 500 at 100 N), elongation was 3.6 ± 0.9 mm in the Me1 group, 3.5 ± 0.6 mm in the Me2 group, and 4.1 ± 1.0 mm in the magnesium group. No significant differences regarding elongation were found between the groups. Load to failure was 352 ± 115 N in the Me1 group, 373 ± 77 N in the Me2 group, and 449 ± 92 N in the magnesium group, with no significant difference between the groups.

**Conclusion:**

In this study, the magnesium bone staples provided appropriate time-zero biomechanical primary stability in comparison with metal bone staples and may therefore be a feasible alternative for cortical fixation of tendon grafts in knee surgery.

**Clinical Relevance:**

The biodegradability of magnesium bone staples would eliminate the need for later implant removal.

Different techniques are available for fixation of peripheral ligaments in knee surgery, with interference screw fixation being the current gold standard.^9^ However, due to the need of creating a bone tunnel to place an interference screw, tunnel conflicts may arise, especially if multiligamentous reconstructions or concomitant osteotomy is performed.^27,28,31^ An extracortical fixation with bone staples could avoid these problems associated with creating a bone tunnel and possibly reducing complications. Previously, bone staples with spikes were shown to display adequate biomechanical primary stability, presenting an alternative to contemporary fixation modalities, currently used in cortical ligament fixation.^14,23^ However, soft tissue reactions to subcutaneously located bone staples have been described, making implant removal necessary in selected patients.^5,20^ One solution for this may be the development of a mechanically stable and biodegradable bone staple, which resolves the need for implant removal.

Implants made of magnesium and its alloys are biodegradable and known to possess mechanical properties comparable with human cortical bone, making it a promising material for orthopaedic implants.^25^ However, previous generations of magnesium implants had shortcomings, including the unpredictable speed of degradation, which could lead to implant failure before adequate healing of the fixed structures could be obtained.^3,54^ Furthermore, during degradation, hydrogen gas is released, which could lead to osteolysis and formation of gas caverns.^37,45^ To account for these problems, different modifications were proposed, including alloying magnesium with other metals, and surface treatments, such as coating or ceramization, which modify the implant degradation kinetics to be better suited for use as orthopaedic implants.^3,25,29,50^

Various magnesium implants are currently in use.^2,54^ However, to our knowledge, a bone staple for soft tissue fixation is not available. Therefore, the goal of this study was to develop a bone staple made from a magnesium alloy, to evaluate its biomechanical primary stability, and to compare it with commercially available bone staples made from other metals. It was hypothesized that the newly developed magnesium staple would provide similar primary stability compared with commercially available metal staples.

## Methods

Porcine knees and lower legs were obtained from a local butcher, who confirmed adequate health and comparable age of all specimens. Different types of preproduction models of implants made from magnesium were produced in cooperation with Medical Magnesium GmbH. All other implants and materials were purchased commercially. No ethics approval was required for human or animal cadaveric studies at our institution.

### Development of a Biodegradable Magnesium Staple

**Figure 1.**
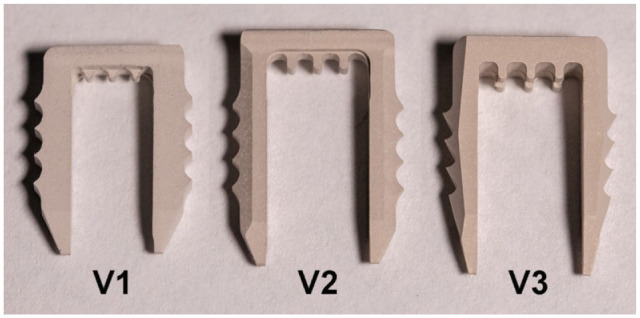
Preproduction variants of magnesium staple prototypes. Different design prototypes of magnesium staples were developed, aiming for favorable balance between fixation stability and implant size before deciding on the final design (V3) to be compared with metal staples.

Development of biodegradable magnesium-based bone staple prototypes was performed in cooperation with Medical Magnesium. Staples were manufactured from rods of solid magnesium alloy on a high-precision lathe. Afterwards, plasma electrolytic oxidation (PEO) was applied as a surface treatment, to further control degradation of the final staple. Different iterations of staple design were developed (V1, V2, V3) (Figure 1) and evaluated regarding their ease of use and biomechanical properties, aiming for a favorable balance between fixation stability and implant size. Staple model V3 was deemed to possess the ideal biomechanical properties for ligament fixation and was used in this study. To create a clinical scenario, the staples were gamma sterilized before testing.

### Staple Fixation

**Figure 2.**
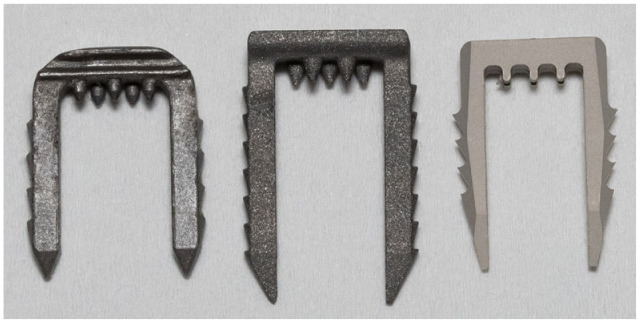
Bone staple models used in this study. From left to right: Richards fixation staple with spikes (Me1; Smith & Nephew); spiked ligament staple (Me2; Arthrex); magnesium staple prototype (Mg; Medical Magnesium). Me1, metal staple 1; Me2, metal staple 2; Mg, magnesium staple.

Different iterations of staple design prototypes were developed (Figure 1) and evaluated biomechanically before deciding on the design utilized in this study. The final magnesium bone staple had a width of 8 mm and a length of 17 mm. The following staple models were commercially purchased: Richards fixation staple with spikes (Smith & Nephew), width 8 mm, length 15 mm, made from cobalt-chrome alloy (metal staple 1 [Me1]) (Figure 2); and spiked ligament staple (Arthrex), width 8 mm, length 20 mm, made from cobalt-chrome alloy (metal staple 2 [Me2]; Figure 2).

**Figure 3.**
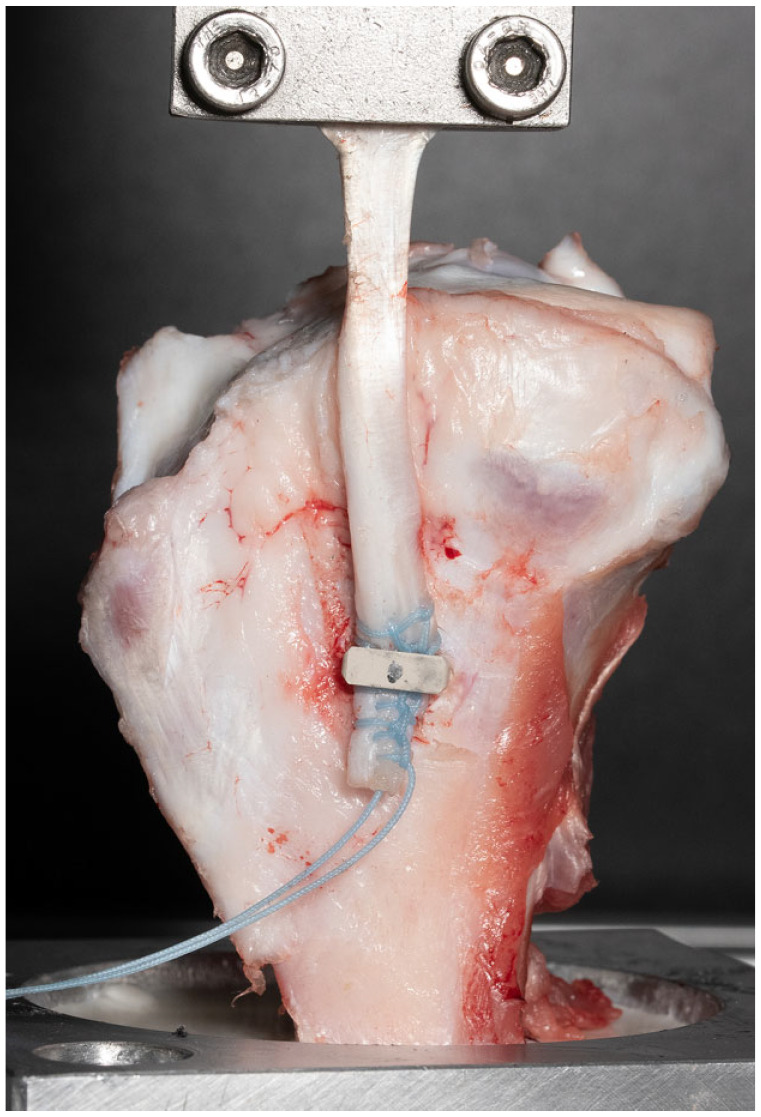
Experimental setup for biomechanical testing. An 8-mm-wide magnesium staple was used to fix a 6-mm-diameter tendon graft, sutured in Krackow technique with high-strength polyethylene suture material to the anatomical MCL insertion site, approximately 40 mm distal to the joint line. MCL, medial collateral ligament.

Tibiae and superficial flexor tendons were prepared from fresh porcine hind legs and were frozen at -20°C. For testing, specimens were defrosted at 7°C for 24 hours and fixed in a cylindrical mount using synthetic resin (RenCast FC 52/53 A ISO and Ren Cast FC 53 B Polyol; Gößl & Pfaff). Superficial flexor tendons were trimmed to a length of 80 mm and a diameter of 6 mm to simulate a tendon graft for medial collateral ligament (MCL) reconstruction. The distal 20 mm of the tendon grafts were sutured in Krackow technique on each side using a high-strength polyethylene suture (No. 2 FiberWire; Arthrex).^38^ The anatomic tibial insertion site of the porcine MCL was identified approximately 40 mm distal to the joint line and the bone exposed. Envelope randomization was utilized to determine the order in which the different staples were tested. The sutured tendon grafts were fixed to the bone by impacting the staple at a 90° angle to the bony surface and the tendon graft (Figure 3).

### Biomechanical Testing

A servohydraulic uniaxial testing machine (Model 8874, Instron) with a 0 to 20 kN sensor was utilized to determine the biomechanical primary stability. The accuracy was ±0.005% (±1 N) of the load cells’ rated output, allowing a position control with an accuracy of ±0.5% of the desired crossbar position. The cylindrical mount containing the embedded porcine tibia was fixed to the base of the machine with 2 clamps. The proximal end of the graft was fixed to the testing machine using a cryoclamp, leaving 20 mm of free graft between the clamp and the joint line. Before testing, the construct was pretensioned manually with a force of 20 N, by positioning of the machine's crossbar.

The following test protocol was applied, as previously described^23^: before the start of cyclic loading, 10 cycles at 50 N were performed for preconditioning of the tendon graft. Cyclic loading was performed at a frequency of 1 Hz. To simulate the forces occurring in the native human MCL in the anterior cruciate ligament (ACL)-intact knee, 500 cycles at 50 N were performed, followed by 500 cycles at 100 N to simulate the forces in the ACL-deficient knee.^40,41,49^ Finally, the construct was loaded continuously at a speed of 25 mm per minute until failure of the construct occurred. Stiffness was calculated from the slope of the linear portion of the load-displacement curve during load to failure. The mode of failure was documented macroscopically.

### Statistical Analysis and Sample Size Calculation

Statistical analysis was performed using Matlab (Version R2020a, MathWorks) and PRISM (Version 8, GraphPad Software). The results are presented as means and standard deviations. The criteria for normal distribution of the data were controlled utilizing histograms as well as the Shapiro-Wilk test. Since not all groups passed the test for normality, the Kruskal-Wallis test was used to compare groups. Post hoc Dunn correction was performed to account for multiple testing. A *P* value of <.05 was deemed the threshold for statistical significance.

An a priori power analysis showed that a sample size of n = 10 per group would yield a 90% power to detect a difference of 50 N between group means at the *f*≥ 0.8 level based on the standard deviations of graft fixation methods in porcine knee models, as reported in previous studies.^24,43^

## Results

After 500 cycles at 50 N, elongation was 1.5 ± 0.5 mm in the Me1 staples, 1.9 ± 0.5 mm in the Me2 staples, and 1.8 ± 0.4 mm in the magnesium staples. After 1000 cycles of loading (500 cycles at 50 and 500 cycles at 100 N), elongation was 3.6 ± 0.9 mm in the Me1 staples, 3.5 ± 0.6 mm in the Me2 staples, and 4.1 ± 1.0 mm in the magnesium staples. No significant differences regarding elongation were found between the groups (*P* > .05).

Load to failure was 352 ± 115 N in the Me1 staples, 373 ± 77 N in the Me2 staples, and 449 ± 92 N in the magnesium staples. No significant differences regarding load to failure were found between the groups (*P* > .05).

**Figure 4.**
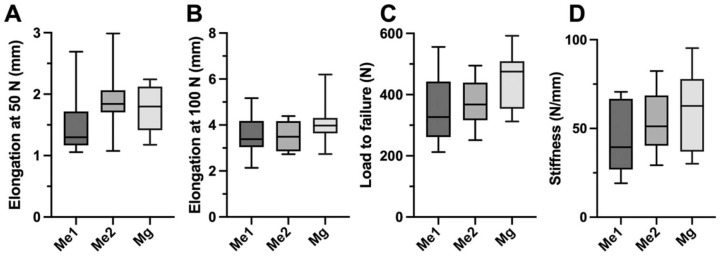
Boxplots representing median (line), interquartile range (box), and range (whiskers) for (A) elongation after cyclic loading at 50 N, (B) cyclic loading at 100 N, (C) load to failure, and (D) stiffness. Me1, metal staple 1 (Richards fixation staple with spikes); Me2, metal staple 2 (spiked ligament staple); Mg, magnesium staple.

Stiffness was 44.2 ± 19.4 N/mm in the Me1 staples, 53.5 ± 16.9 N/mm in the Me2 staples, and 59.6 ± 23.0 N/mm in the magnesium staples. There was no significant difference observable between stiffness of the different staple fixations. The results are summarized in Table 1 and Figure 4A-D.

**Table 1:** Elongation, Load to Failure, and Stiffness According to Staple Model^*a*^

	Me1	Me2	Magnesium
Elongation at 50 N, mm	1.5 ± 0.5	1.9 ± 0.5	1.8 ± 0.4
Elongation at 100 N, mm	3.6 ± 0.9	3.5 ± 0.6	4.1 ± 1.0
Load to failure, N	352 ± 115	373 ± 77	449 ± 92
Stiffness, N/mm	44.2 ± 19.4	53.5 ± 16.9	59.6 ± 23.0

a Data are reported as mean ± SD. Me1, metal staple 1 (Richards fixation staple with spikes), Me2, metal staple 2 (spiked ligament staple).

The mode of failure was tendon pullout in 8 specimens of the Me1 group, 9 specimens of Me2 group, and 6 specimens of the magnesium group. In the remaining specimens, the forces acting on the tendon graft led to a proximal tilt of the staple followed by a pullout from the bone. There were no significant differences in elongation and load-to-failure values between the 3 groups.

## Discussion

The most important finding of this study was that a newly developed bone staple made from magnesium provided similar primary stability compared with bone staples made from metal. Furthermore, magnesium bone staples showed a similar failure mechanism in comparison with metal bone staples.

A 2021 study showed that bone staples displayed comparable primary stability compared with interference screws and superior primary stability compared with suture anchors regarding elongation (3.4 ± 1.0 mm) and load to failure (376 ± 120 N).^23^ A subsequent biomechanical study published in 2022 showed that bone staples without spikes showed significantly inferior primary stability in comparison with bone staples with spikes, irrespective of the width or the number of prongs of the staple, as they were not able to grip the suture material.^14^ In a biomechanical study of MCL reconstruction in a porcine model, it was shown that additional suturing of the graft area gripped by the implant increased primary stability significantly, irrespective of which implant was used.^43^ Owing to these results, we used staples with spikes and comparable width, with additional suturing of the distal end of the tendon graft, in the present study.

Different extracortical fixation strategies have been compared for MCL reconstruction in a biomechanical study, in a porcine model similar to that used in the present study.^43^ A 4.0-mm cancellous screw with a spiked polyether ether ketone washer resulted in the highest primary stability, with elongation during cyclic loading for 250 cycles at 100 N being 2.9 ± 0.7 mm, and ultimate failure load of 469.8 ± 64.3 N. These results are comparable with the values achieved in this study and indicate that bone staples, made from both metal and magnesium, could be an alternative to other extracortical fixation modalities used currently.

Data from computer simulations have estimated the peak force of the native MCL during walking to be approximately 34 N.^49^ A 2016 study measured the forces in the native MCL to range around 50 N.^48^ These forces increase in the ACL-deficient knee to approximately 114 N.^49^ Since MCL injuries frequently occur concomitant to an injury of the ACL, cyclic loading was performed with 2 different loads (50 and 100 N) in the current study to simulate both the ACL-intact and -deficient state.^7^

The reported load-to-failure values of the native human MCL range from 465 ± 190 N to 534 ± 55 N.^35,47,53^ Although load to failure of the tested staples in the current study was lower than the values reported for the native MCL, loading of the graft to failure during rehabilitation is not expected. All the bone staples tested in this study were able to withstand cyclic loading without premature failure indicating that all of them are biomechanically suitable for reconstruction of the MCL.

Bone staples are currently used for various indications in knee surgery. Their use for graft fixation in MCL reconstruction was described previously in noncontrolled clinical studies, with acceptable or good clinical outcome.^18,26,30^ Recent systematic reviews examining repair and reconstruction techniques of the MCL, including fixation with bone staples, were not able to find clinical inferiority of bone staple fixation in comparison with interference screw or suture anchor fixation.^15,16^ Another important use is the fixation of a strip of the distal iliotibial band to the femur, in anterolateral tenodesis of the knee.^10,11,13,20,32,44,56^ Although a rather low rate of implant irritation is reported when using bone staples, it can still occur and make a second surgery necessary.^5,11^ The STABILITY I controlled trial, which used metal staples for fixation of an iliotibial band strip in anterolateral tenodesis, reported the necessity of hardware removal in 3% of included patients.^21^ Furthermore, breakage of the tibial bone is reported when trying to extract bone staples from cortical bone.^22^ This can be avoided if using a biodegradable material. In a recent study, staples were found to frequently conflict with the femoral tunnel of an ACL reconstruction, possibly impairing the integrity of the graft; however, that study used bone staples with a length of 25 mm.^39^ The longest staples utilized in the current study were 20 mm in length, the shortest 15 mm. By using a staple of shorter length, the possibility for a conflict with the femoral ACL tunnel may be reduced, although not excluded entirely.

In addition to adequate biomechanical stability, which was shown in this study, magnesium possesses additional beneficial characteristics. The mechanical properties of magnesium are closer to human cortical and spongious bone than other biodegradable materials or titanium.^25^ Magnesium was shown to possess osteoinductive effects, improving both bone growth and fracture healing, both in vitro and in small-animal models.^55,57,58^ Furthermore, magnesium might improve tendon-bone healing during ligament reconstruction. Cheng et al^12^ showed that an ACL reconstruction in a rabbit model fixed with interference screw made from high purity magnesium (hp-Mg-IFS) showed significantly superior biomechanical stability in comparison with titanium screws during the course of healing. Wang et al^51,52^ found that hp-Mg-IFS improved the speed of the tendon integration into an extra-articular bone tunnel in comparison with titanium screws.

Previous implants made from magnesium and its alloys, however, suffered from certain drawbacks. Magnesium degrades by corrosion when in contact with aqueous solutions,^54^ releasing hydrogen gas, which could possibly lead to the formation of intraosseous osteolysis and gas caverns if released at high dose levels.^37,45^ Unpredictable speed of degradation could lead to implant failure before adequate healing of the fixed structures is be obtained.^3^ Different approaches were introduced to address these problems, including alloying magnesium with other metals, as well as surface modifications.^3,34,50^ The ligament staple prototypes (Medical Magnesium GmbH) used in this study were made from magnesium alloy WE43MEO, which contains yttrium, zirconium, and rare earth elements. WE43 alloys were shown previously to begin degradation between weeks 12 and 16 in a small animal model,^33^ and they exhibited favorable degradation kinetics in comparison with poly-l-lactic acid implants.^36^ PEO coating, as used with the tested staples, was shown to decrease the degradation rate and hydrogen gas release in fluid immersion experiments.^4^

### Limitations

This biomechanical study has several limitations to mention. First, this was a time-zero biomechanical study. The speed of degradation, and how this affects the stability of the magnesium staple during the postoperative period, as well as possible influences of the staple on ligament-bone healing, have not been assessed and may be included in future in vivo studies. Furthermore, changes of the material properties of the staples after cyclic loading and loading to failure were not performed. The anatomy of porcine knees was shown to be sufficiently similar to that of human knees,^46^ and porcine flexor tendons possess biomechanical properties similar to those of human hamstring tendons,^17^ making it a frequently used model to assess the biomechanical primary stability of orthopaedic implants.^19,23,24,43^ Nevertheless, bone density in the porcine model is significantly higher in comparison with humans,^1^ which could have affected the findings of the present study.^8,42^ A recent study showed that, in a test setup like that used in this study, the displacement of the machine actuator used to calculate the elongation was significantly higher than the actual graft slippage, as measured by optical tracking at the tunnel aperture, although both values were highly correlated.^6^ Therefore, the test setup used in this study was sufficient to compare different implant types. However, transfer to the clinical setting should be done with caution.

As this was a time-zero study, the influence of the different materials on tendon-bone healing could not be evaluated. Furthermore, the staples used in this study had different geometries, which could have possibly influenced their biomechanical properties. However, different staple geometries have been compared previously, with no statistically significant differences between different designs, if spikes were present.^14^ When magnesium is used for orthopaedic implants, different types of magnesium and surface modifications are available. Possible influences of these differences on the in vivo results of the study could not be assessed.

## Conclusion

In the current study, bone staples made from magnesium displayed time-zero primary stability comparable with conventional metal bone staples and may therefore be a possible alternative for cortical fixation of ligament grafts.
